# Treatment of Fractured Teeth

**Published:** 1876-12

**Authors:** 


*Treatment of a Fractured Teeth.*—Dr. E. A. Bogue, of the New York Odontological Society, recently described an ingenious operation, which he performed in the case of an incisor which had been split vertically, allowing the anterior and posterior plates of enamel to stand alone. The cavity ran from one approximal surface over the cutting edge to the other approximal surface, and, after trying various means of plastering the tooth together, he at last drilled a hole from front to rear, through, and countersunk that on the labial side which sufficed to accomodate the screw head which he intended to use. The hole on the lingual side he made conical and formed a wooden peg that fitted it accurately. Then by means of a drill the same size as the screw, he drilled a hole into the plug. At every turn of the screw the two pieces of the tooth were drawn closer together, the countersink in the labial surface holding the head of the gold, and the wooden nut upon the lingual side being conical, tended constantly toward the head of the screw as this latter was turned. The cavity was filled with the oxychloride of zinc.

An operation somewhat similar was performed by Dr. S. H. Williams, of this city. The fractured tooth was a superior bicuspid from which the labial half had been altogether detached. By means of a gold wire passing from the labial to the palatine surface and through the detached part, this latter was firmly attached to the portion of crown remaining on roots, and the space which was in the greater part formed by two proximal cavities anterior and posterior, and which had, before the accident been filled, was built up with gold, making a beautiful operation.

